# Serum vitamin E level and functional prognosis after traumatic brain injury with intracranial injury: A multicenter prospective study

**DOI:** 10.3389/fneur.2022.1008717

**Published:** 2022-10-19

**Authors:** Gwan Jin Park, Young Sun Ro, Hanna Yoon, Stephen Gyung Won Lee, Eujene Jung, Sung Bae Moon, Sang Chul Kim, Sang Do Shin, Pan-Asia Trauma Outcomes Study for Traumatic Brain Injury (PATOS-TBI) research network

**Affiliations:** ^1^Department of Emergency Medicine, Chungbuk National University Hospital, Cheongju, Republic of Korea; ^2^Laboratory of Emergency Medical Services, Seoul National University Hospital Biomedical Research Institute, Seoul, Republic of Korea; ^3^Department of Emergency Medicine, Seoul National University Hospital, Seoul, Republic of Korea; ^4^Department of Emergency Medicine, Seoul National University College of Medicine, Seoul, Republic of Korea; ^5^Department of Emergency Medicine, Seoul National University Boramae Medical Center, Seoul, Republic of Korea; ^6^Department of Emergency Medicine, Chonnam National University Hospital, Gwangju, Republic of Korea; ^7^Department of Emergency Medicine, School of Medicine Kyungpook National University and Kyungpook National University Hospital, Daegu, Republic of Korea

**Keywords:** vitamin E, prognosis, biomarker, traumatic brain injury, trauma

## Abstract

**Background:**

Traumatic brain injury (TBI) is a major public health problem with high mortality and disability. Vitamin E, one of the antioxidants for treatment of TBI, has not been sufficiently evaluated for predicting prognosis of TBI. This study aimed to evaluate the prognostic value of vitamin E on functional outcomes of TBI patients with intracranial injury.

**Methods:**

A multi-center prospective cohort study was conducted in five university hospitals between 2018 and 2020. Adult TBI patients who visited the emergency department (ED) with intracranial hemorrhage or diffuse axonal injury confirmed by radiological examination were eligible. Serum vitamin E levels (mg/dL) were categorized into 4 groups: low (0.0–5.4), low-normal (5.5–10.9), high-normal (11.0–16.9), and high (17.0–). Study outcomes were set as 1- and 6-month disability (Glasgow outcome scale (GOS) 1–4). Multilevel logistic regression analysis was conducted to calculate the adjusted odds ratios (AORs) of vitamin E for related outcomes.

**Results:**

Among 550 eligible TBI patients with intracranial injury, the median (IQR) of serum vitamin E was 10.0 (8.0–12.3) mg/dL; 204/550 (37.1%) had 1-month disability and 197/544 (36.1%) had 6-month disability of GOS 1–4. Compared with the high-normal group, the odds of 1-month disability and 6-month disability increased in the low and low-normal group (AORs (95% CIs): 3.66 (1.62–8.27) and 2.60 (1.15–5.85) for the low group and 1.63 (1.08–2.48) and 1.60 (1.04–2.43) for the low-normal group, respectively).

**Conclusion:**

Low serum vitamin E level was associated with poor prognosis at 1 and 6 months after TBI with intracranial injury.

## Introduction

Traumatic brain injury (TBI) is a serious public health burden worldwide, with more than 10 million people worldwide hospitalized or dead annually from TBI (1). TBI occurs frequently in younger age, which results in high mortality or leaving a permanent impairment (2). In addition, the disease burden of TBI is expected to increase further due to aging of the population as well as improvement in immediate life-saving treatment (3, 4).

Vitamin E is a lipid-soluble antioxidant that reduces reactive oxygen species. Vitamin E deficiency causes degeneration of neurons, particularly peripheral axons and posterior column neurons, which results in peripheral neuropathy, ataxia, and skeletal myopathy (5, 6). In the pathophysiology of TBI, increased production of free radicals and reactive oxygen species after injury leads to oxidative stress and secondary neurotoxicity (7). In cases where there is a deficiency of various antioxidants, involved in recovery of brain tissue post injury, treatment with antioxidants may be theoretically effective in preventing propagation of tissue damage as well as in improving short- and long-term survival/functional outcomes (8–12). Several animal studies have evaluated that vitamin E supplementation before TBI reduced oxidative stress and improved learning and memory (13, 14). Vitamin E administration after TBI also reduced microscopic brain damage, promoted nerve regeneration, and improved cognitive function in animal models (15–17). In clinical settings, early antioxidant supplementation including vitamin E in critically ill patients, including TBI patients, was associated with decrease in organ failure and hospital stay (18, 19). Vitamin E supplementation showed a significant reduction in mortality and improvement of long-term functional outcomes of TBI patients (20).

Given the evidence of the beneficial effects of vitamin E, it was hypothesized that vitamin E deficiency would be associated with poor survival outcomes and functional recovery after TBI with intracranial injury, and that serum vitamin E levels could be utilized as a nutritional biomarker for clinical outcomes after TBI. This study aimed to determine the association between serum vitamin E levels and functional/survival outcomes among TBI patients with intracranial injury.

## Methods

### Study design, setting, data source

This was a multi-center prospective cohort study conducted in five participating university hospitals in Korea based on the Pan-Asian Trauma Outcome Study (PATOS) registry (21). The PATOS-TBI study is a collaborative research network that started in 2018 throughout Korea for in-depth research on TBI (ClinicalTrials.gov, ID: NCT04718935). The objectives of PATOS-TBI study are to identify nutritional and metabolic biomarkers related to prognosis of TBI with intracranial injury, and to develop a prognostic predictive model of long-term prognosis that applies them to select high-risk populations funded by the National Research Foundation of Korea (22).

The inclusion criteria were TBI patients with intracranial injury confirmed by a brain radiological examination. Intracranial injury was defined as intracranial hemorrhage or diffuse axial injury (ICD-10 S06.1–06.9). Patients with TBI over 18 years of age who visited participating hospitals' ED using emergency medical services (EMS) within 72 h of injury were enrolled. Patients with penetrating brain injury, history of psychiatric or neurological disorders, terminal cancer, pregnant women, and those transferred after surgery from other hospitals were excluded from the study.

The PATOS-TBI study registry comprises of several variables including the patient's demographics, injury-related information, emergency medical service (EMS) records, clinical findings, laboratory test results, brain imaging findings, diagnoses and medical treatment in hospitals, and patient outcomes at time of hospital discharge and follow-up.

Upon confirmation of intracranial injury on brain computer tomography (CT) and magnetic resonance imaging (MRI), an emergency physician obtained informed consent for enrollment and registered the patients to the PATOS-TBI study during the ED treatment process and blood samples were obtained for biomarker analysis. Based on the informed consent from the patient or guardians, primary surveillance data were collected by an emergency physician. A trained research coordinator from each participating hospital collected and entered the registry through interviews and electronic medical records review. Serum biomarker levels were not reported to physicians, and patient management was not altered by the study. Follow-up data at 1- and 6-months after the injury were captured *via* telephone surveys. All research coordinators were required to receive education and training prior to participation in the study and periodically during the study period. Data were collected using a standardized data collection protocol, a case report form, and a web-based data collection system. The Quality Management Committee (QMC) reviewed the data monthly and provided regular feedback for quality assurance (22, 23).

### Study population

The study population included all adult TBI patients who visited the participating EDs between 2018 and 2020 with intracranial injury (intracranial hemorrhage or diffuse axonal injury, ICD-10 S06.1–06.9) confirmed by brain CT or MRI. Patients with unknown information regarding vitamin E levels and 1-month GOS score were excluded.

### Main outcomes

The primary outcome was 1-month disability after injury, which was defined as Glasgow Outcome Scale (GOS) score of 1 to 4. GOS was scored from 0 to 5 as follows: 1 (dead), 2 (vegetative state), 3 (severe disability), 4 (moderate disability), and 5 (good recovery) (24). The secondary outcome was 1-month mortality and the tertiary outcomes were 6-month disability and 6-month mortality.

### Analysis of serum biomarkers

Upon confirmation of intracranial injury on brain CT or MRI and patients' consent for study enrollment, 24 mL of blood was drawn *via* venipuncture in the ED. Centrifugation was performed at 3,000 rpm for 10 min at room temperature within an h of blood sampling. Levels of serum α-tocopherol, the most biologically active form of vitamin E, were analyzed. Vitamin E is a collective term that refers to eight fat-soluble forms with antioxidant activities. The liver preferentially re-secretes only α-tocopherol *via* the α-tocopherol transport protein and degrades all other forms of vitamin E. Therefore, α-tocopherol is the most recognized form of vitamin E in human body, and most research on vitamin E is performed using α-tocopherol level (25).

Serum samples were kept frozen at −20°C and were analyzed within 7 days for serum biomarkers. After dispensing 500 μL of calibrator and dispensing the sample into a 15 mL conical tube, six calibration standard solutions were prepared for each standard concentration. The solutions were adjusted to the total volume of 500 μL, with addition of 1 mL of ethanol for deproteinization. Next, 3 mL of N-Hexane was added, then centrifuging at 3,000 rpm for 5 min. After transferring 2 mL of the supernatant to the test tube and evaporating with nitrogen gas, 150 μL of methanol was added to the residue and transferred to a vial. These pretreated samples were analyzed after standard testing with High-performance liquid chromatography (HPLC).

### Variables and measurements

Considering the normal range of vitamin E level in adults, the plasma vitamin E level was categorized into four groups: low (0.0–5.4 mg/dL), low-normal (5.5–10.9 mg/dL), high-normal (11.0–16.9 mg/dL), and high group (17.0– mg/dL) (26).

Information were collected including the patients' demographics (age, sex, education, comorbidities, body mass index, and pre-injury disability), injury characteristics (mechanism of injury (road traffic injury, fall, blunt trauma, and others), date and time of injury, and alcohol intake at injury, ED (Glasgow coma scale score at arrival to the ED, transfer from other hospitals, time interval from injury to ED, type of intracranial injury on brain CT or MRI, injury severity, and length of stay in ED), and outcomes (ED disposition and hospital outcome, 1-month and 6-month follow up outcomes).

### Statistical analysis

Descriptive analysis was conducted to compare the characteristics of study population according to serum vitamin E levels. Categorical variables were reported as counts and proportions and continuous variables were reported as medians and interquartile ranges. Differences between groups were compared using Pearson's chi-square test and Wilcoxon rank sum test.

Adjusted odds ratios (AORs) with 95% confidence intervals (CIs) were calculated using multilevel multivariable logistic regression analysis to examine associations between serum vitamin E levels and study outcomes in TBI patients after adjusting for hospital clustering. Potential confounders were selected based on directed acyclic graph (DAG) models and included age, sex, obesity (body mass index ≥25), high education (college and more), comorbidities (hypertension and diabetes mellitus), pre-injury disability (mRS score 3–5), and injury severity (AIS score of TBI ≥3). Variables were adjusted in the model as potential confounders if they block all back-door paths from the main exposure to the outcome in DAG models (Supplementary Figure 1); affect the outcome; and are not mediators in a causal pathway (27, 28). Akaike information criterion (AIC) and Bayesian information criterion (BIC) values were calculated to evaluate a goodness-of-fit of the model.

Receiver operating characteristic (ROC) analysis was performed to evaluate the performance of serum vitamin E levels and Youden's Index was used to find the best cut-off point of serum vitamin E levels in predicting study outcomes.

A two-sided *P* < 0.05 was defined as significant. All statistical analyses were performed using SAS software, version 9.4 (SAS Institute Inc., Cary, NC, USA).

### Ethics statement

The study was approved by the Institutional Review Board of all participating hospitals (IRB no.: SNUH-1806-078-951; CNUH-2018-297; KNUH-2018-10-014-007; CBNUH-2018-09-018; BMC-30-2018-85). Informed consent was obtained from the patients or family members/legal representatives of unconscious patients. Patient information was anonymized prior to analysis.

## Results

A total 550 adult TBI patients out of 606 patients were included in this study, excluding 56 patients for whom information on 1-month GOS was unknown.

Table 1 presents the demographics of TBI patients according to the vitamin E levels. The median (IQR) of serum vitamin E was 10.0 (8.0–12.3) mg/dL and vitamin E deficiency was observed in 35/550 (6.4%) of the study population. The proportion of 1-month disability after injury was 37.1% (204/550) in study population, and 1-month mortality was 18.7% (103/550). Among 544 patients who were followed up to 6-months after injury, 36.1% (197/544) had disability of GOS 1–4 and 20.5% (112/544) had mortality. The proportions of 1-month disability after injury were 65.7% (23/35) for the low group, 40.5% (123/304) for the low-normal group, 27.3% (50/183) for the high-normal group, and 28.6% (8/28) for the high group, respectively (*p* < 0.001).

**Table 1:** Demographics of study patients according to the serum vitamin E levels.

	**Total**		**Serum vitamin E level (mg/dL)**								
			**Low (0.0–5.4)**		**Low-normal (5.5–10.9)**		**High-normal (11.0–16.9)**		**High (17.0–)**		
	**N**	**%**	**N**	**%**	**N**	**%**	**N**	**%**	**N**	**%**	***P*–value**
Total	550		35		304		183		28		
Sex, female	173	31.5	6	17.1	89	29.3	68	37.2	10	35.7	0.072
Age, year											0.673
18–60	173	31.5	9	25.7	101	33.2	57	31.1	6	21.4	
60–80	285	51.8	19	54.3	152	50.0	95	51.9	19	67.9	
80–120	92	16.7	7	20.0	51	16.8	31	16.9	3	10.7	
Median (IQR)	68 (56–77)		72 (59–79)		68 (55–77)		65 (56–77)		72 (62–76)		0.353
Education											0.087
High school or less	398	72.4	27	77.1	220	72.4	129	70.5	22	78.6	
College or more	100	18.2	1	2.9	58	19.1	38	20.8	3	10.7	
Others	52	9.5	7	20.0	26	8.6	16	8.7	3	10.7	
Pre-injury disability, mRS 3–5	43	8.0	5	14.3	27	8.9	7	4.4	4	14.3	0.165
Underlying disease											
Hypertension	216	39.3	10	28.6	125	41.1	69	37.7	12	42.9	0.484
Diabetes mellitus	141	25.6	8	22.9	85	28.0	40	21.9	8	28.6	0.475
Chronic liver disease	25	4.5	5	14.3	11	3.6	7	3.8	2	7.1	0.325
Chronic kidney disease	34	6.2	1	2.9	23	7.6	10	5.5	0	0.0	0.348
Body mass index, ≥ 25 kg/m^2^	117	21.3	5	14.3	70	23.0	31	16.9	11	39.3	0.029
Day of injury, weekend	162	29.5	10	28.6	89	29.3	57	31.1	6	21.4	0.768
ED visit time, nighttime	215	39.1	15	42.9	115	37.8	77	42.1	8	28.6	0.494
Alcohol intake at injury	50	9.1	2	5.7	32	10.5	15	8.2	1	3.6	0.484
Mechanism											0.057
Road traffic injury	223	40.5	17	48.6	128	42.1	69	37.7	9	32.1	
Fall	239	43.5	14	40.0	136	44.7	78	42.6	11	39.3	
Blunt trauma	69	12.5	1	2.9	30	9.9	30	16.4	8	28.6	
Others	19	3.5	3	8.6	10	3.3	6	3.3	0	0.0	
Transfer from other hospitals	242	44.0	18	51.4	128	42.1	82	44.8	14	50.0	0.640
Glasgow coma scale score											< 0.001
3–8	126	22.9	17	48.6	68	22.4	37	20.2	4	14.3	
9–12	52	9.5	4	11.4	32	10.5	14	7.7	2	7.1	
13–15	372	67.6	14	40.0	204	67.1	132	72.1	22	78.6	
Time interval from injury											
to ED, hour, median (IQR)	1.7 (0.7–3.3)		1.6 (0.8–3.1)		2.0 (0.7–3.7)		1.3 (0.6–3.0)		1.7 (0.6–3.1)		0.307
to blood sampling, hour, median (IQR)	0.6 (0.5–0.7)		0.6 (0.5–0.7)		0.6 (0.5–0.8)		0.6 (0.5–0.6)		0.6 (0.5–0.8)		0.785
Type of intracranial injury											
Diffuse axonal injury	36	6.5	2	5.7	20	6.6	14	7.7	0	0.0	0.732
Intracerebral hemorrhage	121	22.0	3	8.6	70	23.0	43	23.5	5	17.9	0.223
Subarachnoid hemorrhage	216	39.3	11	31.4	123	40.5	70	38.3	12	42.9	0.726
Subdural hemorrhage	411	74.7	28	80.0	225	74.0	139	76.0	19	67.9	0.695
Epidural hemorrhage	80	14.5	2	5.7	47	15.5	27	14.8	4	14.3	0.492
Intraventricular hemorrhage	47	8.5	5	14.3	28	9.2	12	6.6	2	7.1	0.149
Injury severity											
AIS score of TBI, 3–6	462	84.0	32	91.4	258	84.9	151	82.5	21	75.0	0.309
ISS, median (IQR)	17 (10–25)		20 (10–27)		17 (10–25)		16 (9–22)		13 (9–22)		< 0.001
ED length of stay, hour, median (IQR)	3.1 (2.0–5.4)		3.9 (2.4–5.5)		2.9 (1.9–5.3)		3.1 (1.9–5.4)		3.5 (2.1–7.1)		0.665
Outcomes											
1-month disability, GOS 1–4	204	37.1	23	65.7	123	40.5	50	27.3	8	28.6	< 0.001
GOS 1	103	18.7	16	45.7	66	21.7	18	9.8	3	10.7	< 0.001
GOS 2	8	1.5	3	8.6	2	0.7	2	1.1	1	3.6	
GOS 3	50	9.1	2	5.7	27	8.9	20	10.9	1	3.6	
GOS 4	43	7.8	2	5.7	28	9.2	10	5.5	3	10.7	
GOS 5	346	62.9	12	34.3	181	59.5	133	72.7	20	71.4	
1-month mortality	103	18.7	16	45.7	66	21.7	18	9.8	3	10.7	< 0.001
6-month disability, GOS 1–4 (n=544)	197	36.1	20	58.8	120	39.5	49	27.1	8	29.6	< 0.001
6-month mortality (n=544)	112	20.5	16	47.1	72	23.7	21	11.6	3	11.1	< 0.001

IQR, interquartile range; ED, emergency department; mRS, modified Rankin Scale; TBI, traumatic brain injury; AIS, abbreviated injury scale; ISS, injury severity score; GOS, Glasgow outcome scale.

Table 2 shows the characteristics of TBI patients by 1-month disability. The medians (IQR) of serum vitamin E levels were 9.2 (7.3–11.4) mg/dL for 204 patients who had GOS 1–4 at 1-month after injury and 10.5 (8.6–12.5) mg/dL for 346 patients with GOS 5 (*p* < 0.001).

**Table 2:** Characteristics of study population according to 1-month Glasgow outcome scale score.

	**Total**		**GOS 1–4**		**GOS 5**		**p-value**
	**N**	**%**	**N**	**%**	**N**	**%**	
Total	550		204	37.1	346	62.9	
Vitamin E, mg/dL, median (IQR)	10.0 (8.0–12.3)		9.2 (7.3–11.4)		10.5 (8.6–12.5)		< 0.001
Sex, female	173	31.5	55	27.0	118	34.1	0.081
Age, year, median (IQR)	68 (56–77)		72 (57–79)		65 (55–75)		0.004
Education							0.007
High school or less	398	72.4	145	71.1	253	73.1	
College or more	100	18.2	30	14.7	70	20.2	
Others	52	9.5	29	14.2	23	6.6	
Pre-injury disability, mRS 3–5	43	7.8	31	15.2	12	3.5	< 0.001
Underlying disease							
Hypertension	216	39.3	80	39.2	136	39.3	0.983
Diabetes mellitus	141	25.6	53	26.0	88	25.4	0.887
Chronic liver disease	25	4.5	8	3.9	17	4.9	0.590
Chronic kidney disease	34	6.2	11	5.4	23	6.6	0.555
Body mass index, ≥25 kg/m^2^	117	21.3	50	24.5	67	19.4	0.154
Day of injury, weekend	162	29.5	61	29.9	101	29.2	0.860
ED visit time, nighttime	215	39.1	76	37.3	139	40.2	0.498
Alcohol intake at injury	50	9.1	13	6.4	37	10.7	0.089
Mechanism							0.748
Road traffic injury	223	40.5	85	41.7	138	39.9	
Fall	239	43.5	85	41.7	154	44.5	
Blunt trauma	69	12.5	25	12.3	44	12.7	
Others	19	3.5	9	4.4	10	2.9	
Transfer from other hospitals	242	44.0	98	48.0	144	41.6	0.143
Glasgow coma scale score							< 0.001
3–8	126	22.9	112	54.9	14	4.0	
9–12	52	9.5	34	16.7	18	5.2	
13–15	372	67.6	58	28.4	314	90.8	
Type of intracranial injury							
Diffuse axonal injury	36	6.5	18	8.8	18	5.2	0.097
Intracerebral hemorrhage	121	22.0	52	25.5	69	19.9	0.129
Subarachnoid hemorrhage	216	39.3	82	40.2	134	38.7	0.734
Subdural hemorrhage	411	74.7	162	79.4	249	72.0	0.052
Epidural hemorrhage	80	14.5	22	10.8	58	16.8	0.055
Intraventricular hemorrhage	47	8.5	20	9.8	27	7.8	0.418
AIS score of TBI, ≥3	462	84.0	181	88.7	281	81.2	0.020
Injury severity score, median (IQR)	17 (10–25)		22 (14–25)		16 (9–22)		< 0.001

GOS, Glasgow outcome scale; IQR, interquartile range; ED, emergency department; mRS, modified Rankin Scale; TBI, traumatic brain injury; AIS, abbreviated injury scale.

Table 3 presents the associations between serum vitamin E levels and study outcomes. In the multilevel logistic regression analysis, patients with low vitamin E had higher odds of disability and mortality. The adjusted odds for 1-month disability increased in the low-normal and the low groups when compared with the high-normal group (adjusted ORs (95% CIs): 3.66 (1.62–8.27) for the low group and 1.63 (1.08–2.48) for the low-normal group). Regarding 1-month mortality, 6-month disability, and 6-month mortality, the trends were similar (adjusted ORs (95% CIs): 4.77 (1.96–11.62) and 2.31 (1.28–4.15) for 1-month mortality, 2.60 (1.15–5.85) and 1.60 (1.04–2.43) for 6-month disability, and 4.91 (1.98–12.17) and 2.36 (1.34–4.15) for 6-month mortality, respectively).

**Table 3:** Multilevel logistic regression analysis between serum vitamin E levels and study outcomes.

	**Total**	**Outcomes**		**Model 1**	**Model 2**	**Model 3**
	***N***	***N***	**%**	**OR (95% CI)**	**aOR (95% CI)**	**aOR (95% CI)**
1-month disability, GOS 1–4					
Vitamin E level	550	204	37.1			
Low (0.0–5.4 mg/dL)	35	23	65.7	5.10 (2.36–11.03)	3.76 (1.67–8.47)	3.66 (1.62–8.27)
Low-normal (5.5–10.9 mg/dL)	304	123	40.5	1.81 (1.21–2.69)	1.65 (1.09–2.50)	1.63 (1.08–2.48)
High-normal (11.0–16.9 mg/dL)	183	50	27.3	Ref	Ref	Ref
High (17.0– mg/dL)	28	8	28.6	1.06 (0.44–2.58)	0.73 (0.28–1.91)	0.75 (0.29–1.99)
AIC / BIC				711.4 / 728.7	692.0 / 756.7	690.1 / 759.1
1-month mortality					
Vitamin E level	550	103	18.7			
Low (0.0–5.4 mg/dL)	35	16	45.7	8.71 (3.71–20.45)	5.44 (2.19–13.53)	4.77 (1.96–11.62)
Low-normal (5.5–10.9 mg/dL)	304	66	21.7	2.71 (1.54–4.77)	2.47 (1.36–4.47)	2.31 (1.28–4.15)
High-normal (11.0–16.9 mg/dL)	183	18	9.8	Ref	Ref	Ref
High (17.0– mg/dL)	28	3	10.7	1.16 (0.31–4.28)	0.68 (0.16–2.92)	0.66 (0.16–2.81)
AIC / BIC				501.4 / 499.5	479.9 / 473.6	483.6 / 552.5
6-month disability, GOS 1–4					
Vitamin E level	544	197	36.2			
Low (0.0–5.4 mg/dL)	34	20	58.8	3.82 (1.79–8.16)	2.67 (1.19–6.00)	2.60 (1.15–5.85)
Low-normal (5.5–10.9 mg/dL)	303	120	39.6	1.75 (1.17–2.62)	1.61 (1.06–2.45)	1.60 (1.04–2.43)
High-normal (11.0–16.9 mg/dL)	180	49	27.2	Ref	Ref	Ref
High (17.0– mg/dL)	27	8	29.6	1.13 (0.46–2.74)	0.77 (0.29–2.04)	0.80 (0.30–2.12)
AIC / BIC				704.5 / 721.7	682.7 / 747.2	681.7 / 750.5
6-month mortality						
Vitamin E level	544	112	20.6			
Low (0.0–5.4 mg/dL)	34	16	47.1	7.75 (3.32–18.07)	5.08 (2.05–12.59)	4.91 (1.98–12.17)
Low-normal (5.5–10.9 mg/dL)	303	72	23.8	2.56 (1.50–4.38)	2.39 (1.36–4.20)	2.36 (1.34–4.15)
High-normal (11.0–16.9 mg/dL)	180	21	11.7	Ref	Ref	Ref
High (17.0– mg/dL)	27	3	11.1	0.98 (0.27–3.61)	0.57 (0.14–2.37)	0.56 (0.13–2.38)
AIC / BIC	27	3	11.1	522.0 / 520.0	503.0 / 496.7	502.3 / 495.6

Model 1: unadjusted.

Model 2: adjusted for age, sex, obesity, education, comorbidities (hypertension and diabetes mellitus), and pre-injury disability.

Model 3: adjusted for variables in the Model 2, and injury severity (AIS score of TBI, ≥3).

GOS, Glasgow outcome scale; aOR, adjusted odds ratio; CI, confidence interval; AIC, Akaike information criterion; BIC, Bayesian information criterion; AIS, abbreviated injury scale; TBI, traumatic brain injury.

**Figure 1:**
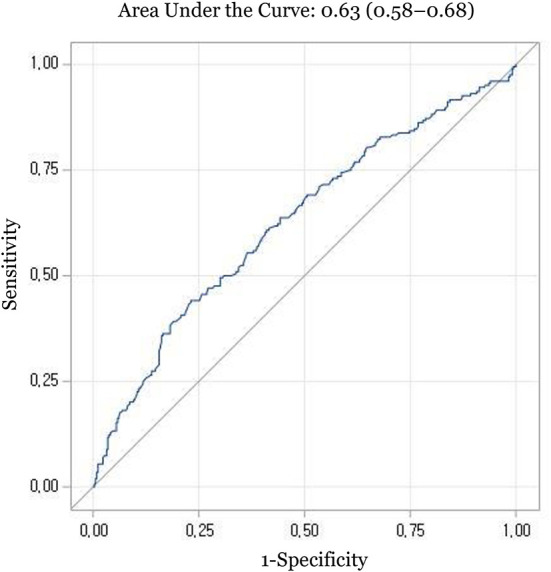
Receiver operating characteristic curve for prediction of 1-month disability with serum vitamin E levels.

In the ROC analysis, the area under the curve (AUC) of serum vitamin E was 0.63 (95% CIs, 0.58–0.68) (Figure 1). The optimal cut-off value of vitamin E was 8.4 mg/dL, corresponding to a sensitivity of 44.0% and a specificity of 76.9%. Serum vitamin E levels < 8.4 mg/dL were significantly associated with poorer outcomes (adjusted ORs (95% CIs): 2.69 (1.82–3.97) for 1-month disability, 2.49 (1.53–4.07) for 1-month mortality, 2.37 (1.57–3.57) for 6-month disability, and 2.54 (1.57–4.10) for 6-month mortality, respectively) (Table 4).

**Table 4:** Association between serum vitamin E levels (binary) and study outcomes.

	**Total**	**Outcomes**		**Model 1**	**Model 2**	**Model 3**
	***N***	***N***	**%**	**OR (95% CI)**	**aOR (95% CI)**	**aOR (95% CI)**
1-month disability, GOS 1–4						
Serum vitamin E level	550	204	37.1			
0.0–8.3 mg/dL	166	88	53.0	2.61 (1.79–3.79)	2.54 (1.71–3.79)	2.69 (1.82–3.97)
8.4– mg/dL	384	116	30.2	Ref	Ref	Ref
AIC / BIC				704.0 / 712.6	681.9 / 737.9	679.9 / 740.2
1-month mortality						
Serum vitamin E level	550	103	18.7			
0.0–8.3 mg/dL	166	50	30.1	2.93 (1.86–4.62)	2.54 (1.56–4.13)	2.49 (1.53–4.07)
8.4– mg/dL	384	53	13.8	Ref	Ref	Ref
AIC / BIC				505.4 / 504.2	481.7 / 476.2	478.0 / 472.2
6-month disability, GOS 1–4						
Serum vitamin E level	544	197	36.2			
0.0–8.3 mg/dL	164	84	51.2	2.53 (1.73–3.70)	2.42 (1.61–3.63)	2.37 (1.57–3.57)
8.4– mg/dL	380	113	29.7	Ref	Ref	Ref
AIC / BIC				695.0 / 693. 8	672.3 / 666.8	671.4 / 665.6
6-month mortality						
Serum vitamin E level	544	112	20.6			
0.0–8.3 mg/dL	164	53	32.3	2.87 (1.84–4.47)	2.58 (1.60–4.15)	2.54 (1.57–4.10)
8.4– mg/dL	380	59	15.5	Ref	Ref	Ref
AIC / BIC				524.5 / 523.4	504.2 / 498.8	503.4 /497.5

Model 1: unadjusted.

Model 2: adjusted for age, sex, obesity, education, comorbidities (hypertension and diabetes mellitus), and pre-injury disability.

Model 3: adjusted for variables in the Model 2, and injury severity (AIS score of TBI, ≥3).

GOS, Glasgow outcome scale; aOR, adjusted odds ratio; CI, confidence interval; AIC, Akaike information criterion; BIC, Bayesian information criterion; AIS, abbreviated injury scale; TBI, traumatic brain injury.

## Discussion

This multi-center prospective cohort study evaluated the prognostic value of serum vitamin E levels on functional/survival outcomes for TBI patients with intracranial injury. There was an association between serum vitamin E levels and 1-month disability (adjusted ORs (95% CIs): 3.66 (1.62–8.27) for the low group and 1.63 (1.08–2.48) for the low-normal group, when compared with the high-normal group). Regarding survival outcomes, there was an association between serum vitamin E levels and 1-month mortality (adjusted ORs (95% CIs): 4.77 (1.96–11.62) for the low group and 2.31 (1.28–4.15) for the low-normal group). These results suggest serum vitamin E as a potential biomarker related to functional and survival outcomes of TBI with intracranial injury.

Reactive oxygen species-induced oxidative damage has been identified as a major contributor to secondary injury from TBI, which has resulted in further damage to neuronal tissue and vasculature. Therefore, various antioxidants such as vitamin B, C, D, zinc, and magnesium have been used before and after TBI to prevent further brain damage (8, 10). Among them, vitamin E has been highlighted because it can easily be implemented in emergency care. It is an inexpensive, safe, and ubiquitous dietary supplement. It is also well tolerated and easily administered *via* enteral feeding (12, 29). Some animal studies have shown the efficacy of vitamin E in exerting a neuroprotective effect by decreasing reactive oxygen species. Vitamin E supplements before and after TBI reduced oxidative stress and showed significant beneficial effects on cognition and mortality (13, 16, 17). Vitamin E is also known as one of the factors inhibiting genetically programmed cell death, and has been used in studies to treat degenerative brain diseases (30). Vitamin E depletion is one of the major factors that increase vulnerability to TBI (8, 9).

The preclinical data supporting vitamin E in TBI are strong. However, only a select few studies have been conducted *in vivo*. Some studies reported that plasma vitamin E levels were rapidly decreased following TBI, which showed early vitamin E depletion and the subsequent need for further supplementation (31, 32). The results of this study also showed similar results, that lower serum levels of vitamin E were associated with poorer outcomes after TBI with intracranial injury. The low and low-normal vitamin E level groups showed significantly poorer survival and functional outcomes. The relative decrease in antioxidant capacity may correlate with the severity of brain injury and suggests a dose-response relationship between vitamin E deficiency and poor neurological outcomes.

Two randomized trials for vitamin E administration in critically ill patients showed only short-term benefits such as reduction of rates of organ failure, inflammatory responses, and a shorter length of stay in the intensive care unit (18, 19). Since the above studies were conducted for critically ill patients, including TBI patients, the interpretation of the results may be limited. The only randomized clinical trial conducted in TBI patients reported that vitamin E administration has showed a beneficial effect in significantly reducing mortality rates and improving functional outcomes at discharge and follow-up (20).

This study is worthwhile in that it prospectively constructed a cohort of TBI patients and identified a relationship between serum vitamin E levels and short-/long-term clinical outcomes after TBI. Currently used diagnostic imaging techniques for TBI are well known to have high initial diagnostic accuracy for intracranial injury, but the clinical role of these on predicting long-term cognitive and physical impairment is limited (33, 34). Based on the results of this study, it can be expected that high-risk groups with poor prognosis will be screened, and the disability and burden of TBI will be reduced through early vitamin E correction and treatment.

This study has several limitations. First, blood samples in this study were collected only at the ED. Serial measurements of vitamin E during hospitalization and understanding the kinetics of vitamin E over time after TBI would be needed to better interpret the findings of this study. Second, potential confounders were selected based on the DAG model. Using the rules of DAG models, the variables in Model 3 were chosen as a minimally sufficient conditioning set to estimate the unbiased effect of serum vitamin E levels. However, there is a possibility that there is some back-door path which can bias the vitamin E levels effects on study outcomes. There may also be unmeasured potential effect modifiers which may violate the assumption of independent effect of vitamin E levels on the study outcome, such as brain volume and anatomical location affected by intracranial injury, and patients' other nutritional status. Third, there were no available data on the history of vitamin E levels before injury, diet diary, and nutritional status, which would have been related to serum vitamin E levels at the ED. Moreover, there were no information about whether vitamin E was supplemented before/after TBI. Fourth, selection bias may arise because the TBI patients with unknown 1-month disability were excluded. Finally, there would be unmeasured and uncontrolled bias when considering the complex pathophysiological processes after TBI. Caution should be taken when interpreting the study results of this study given the significant limitations.

In conclusion, serum vitamin E level is associated with long- and short-term disability and mortality for TBI patients with intracranial hemorrhage and diffuse axonal injury. This study suggests that serum vitamin E may be a potential biomarker in predicting the long- and short-term prognoses of TBI patients with intracranial injury. Further research is needed to evaluate the benefit of vitamin E supplementation in improving the long-term outcomes of TBI patients.

## Data availability statement

The raw data supporting the conclusions of this article will be made available by the authors, without undue reservation.

## Ethics statement

The studies involving human participants were reviewed and approved by of all participating hospitals (Seoul National Univesity Hospital, Seoul National University Boramae Medical Center, Chungbuk National University Hospital, Kyungpook National University Hospital, and Chonnam National University Hospital). Written informed consent to participate in this study was provided by the participants' legal guardian/next of kin.

## Author contributions

Conceptualization, had full access to all of the data in the study, and take responsibility for the integrity of the data as well as the accuracy of the data analysis: GP and YR. Data curation: GP, HY, SL, EJ, and SM. Formal analysis and software, funding acquisition, and writing–original draft: GP. Investigation: YR, HY, SL, EJ, and SM. Methodology: GP, YR, SK, and SS. Supervision: YR, SK, and SS. Validation: HY, SL, EJ, and SM. Visualization: GP and SK. Writing—review and editing: YR and SS. Approval of final manuscript: all authors.

## Funding

This work was supported by the National Research Foundation of Korea (NRF) grant funded by the Korean government (MSIT) (Grant no.: NRF-2018R1C1B6007625 and NRF-2021R1A2C4002898). This funding source had no role in the study design, execution, analyses, interpretation of the data, or decision to submit results.

## Conflict of interest

The authors declare that the research was conducted in the absence of any commercial or financial relationships that could be construed as a potential conflict of interest.

## Publisher's note

All claims expressed in this article are solely those of the authors and do not necessarily represent those of their affiliated organizations, or those of the publisher, the editors and the reviewers. Any product that may be evaluated in this article, or claim that may be made by its manufacturer, is not guaranteed or endorsed by the publisher.
