# Supplementary figures and images for: Serum vitamin E level and functional prognosis after traumatic brain injury with intracranial injury: A multicenter prospective study
Source: Front Neurol. 2022 Oct 19;13:1008717. doi: 10.3389/fneur.2022.1008717 (PMC9627300; doi:10.3389/fneur.2022.1008717)

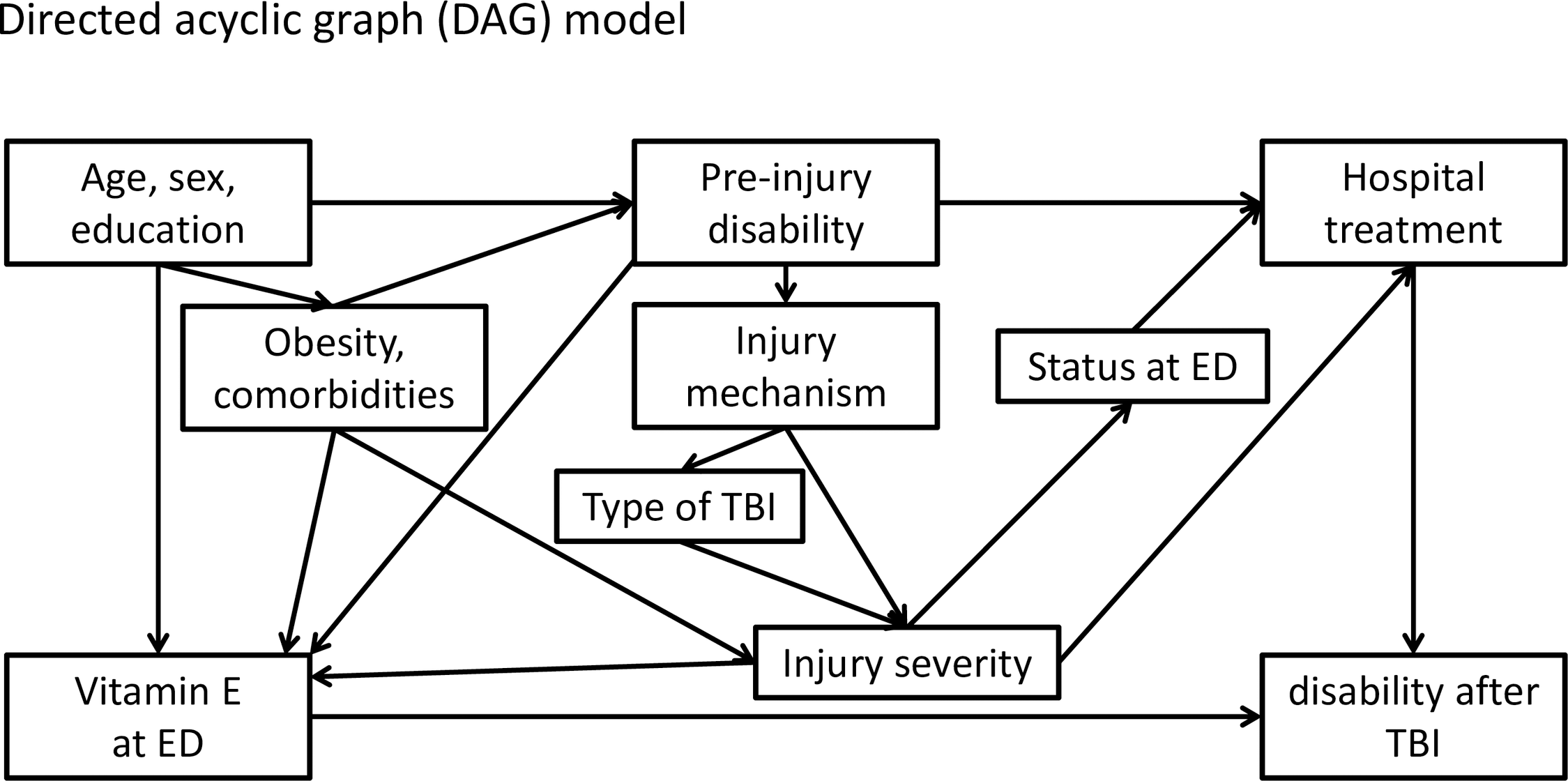

Supplement: Supplementary file 1 [file Image_1.tif]
